# Correlative microscopy and block-face imaging (CoMBI) method for both paraffin-embedded and frozen specimens

**DOI:** 10.1038/s41598-021-92485-5

**Published:** 2021-06-23

**Authors:** Nobukazu Ishii, Yuki Tajika, Tohru Murakami, Josephine Galipon, Hiroyoshi Shirahata, Ryo Mukai, Daisuke Uehara, Ryosuke Kaneko, Yuichi Yamazaki, Yuhei Yoshimoto, Hirohide Iwasaki

**Affiliations:** 1 Department of Anatomy, Gunma University Graduate School of Medicine, Maebashi, Gunma Japan; 2 Department of Neurosurgery, Gunma University Graduate School of Medicine, Maebashi, Gunma Japan; 3 Keio University Institute for Advanced Biosciences, Tsuruoka, Yamagata Japan; 4 Nagoya University Neuroscience Institute of the Graduate School of Science, Nagoya, Japan; 5 Tsuruoka Chuo High School, Tsuruoka, Yamagata Japan; 6 Department of Ophthalmology, Gunma University Graduate School of Medicine, Maebashi, Gunma Japan; 7 Department of Gastroenterology and Hepatology, Gunma University Graduate School of Medicine, Maebashi, Gunma Japan; 8 Bioresource Center, Gunma University Graduate School of Medicine, Maebashi, Gunma Japan

**Keywords:** 3-D reconstruction, Optical imaging, Anatomy

## Abstract

Correlative microscopy and block-face imaging (CoMBI), a method that we previously developed, is characterized by the ability to correlate between serial block-face images as 3-dimensional (3D) datasets and sections as 2-dimensional (2D) microscopic images. CoMBI has been performed for the morphological analyses of various biological specimens, and its use is expanding. However, the conventional CoMBI system utilizes a cryostat, which limits its compatibility to only frozen blocks and the resolution of the block-face image. We developed a new CoMBI system that can be applied to not only frozen blocks but also paraffin blocks, and it has an improved magnification for block-face imaging. The new system, called CoMBI-S, comprises sliding-type sectioning devices and imaging devices, and it conducts block slicing and block-face imaging automatically. Sections can also be collected and processed for microscopy as required. We also developed sample preparation methods for improving the qualities of the block-face images and 3D rendered volumes. We successfully obtained correlative 3D datasets and 2D microscopic images of zebrafish, mice, and fruit flies, which were paraffin-embedded or frozen. In addition, the 3D datasets at the highest magnification could depict a single neuron and bile canaliculus.

## Introduction

Morphological analyses are carried out in various ways in today’s biological laboratories for the purpose of understanding the anatomical structure and molecular distribution in specimens. Traditionally, biological specimens are sliced into sections and observed by light microscopy^1^. Thus, the information obtained by such microscopy is essentially limited to a certain plane in the specimen. To obtain 3-dimensional (3D) anatomical information, researchers have attempted to develop some methods in their laboratories. Serial section microscopy is a method in which a large number of serial sections are collected and used as a volume dataset^2^. However, this approach requires a lot of time to cover the entire specimen, and it is difficult to obtain clear reconstructed planes or volume-rendered images due to distortions of the sections. Block-face imaging is another 3D imaging method in which the exposed block face is serially captured while slicing the block, after which serial block-face images are reconstructed into a 3D image. Block-face imaging can avoid the distortion seen in serial section microscopy. Block-face imaging systems have been developed using light microscope^3,4^ or electron microscope^5^. However, the sections sliced by these block-face imaging systems are discarded and are not used for further microscopic analyses on the distribution of molecules. New devices for 3D imaging, such as X-ray computed tomography (CT) and magnetic resonance imaging (MRI), are now becoming more accessible to biological researchers^6–8^. CT for small animals (µCT) can acquire fine 3D anatomical data from wide varieties of animal species noninvasively. The expanding use of µCT could be due to the development of sample preparation methods, such as iodine staining for contrast enhancement^9^ and freeze-drying of samples^10^. There have been attempts to obtain 3D distribution data of specific molecules using CT and MRI, e.g., the development of probes for molecular imaging and the development of multimodal methods^11,12^. Despite these developments, it is still difficult to label molecules as desired and subsequently image the 3D distribution of molecules using CT or MRI. To depict the 3D distribution of molecules in the laboratory, a light microscope such as a confocal laser scanning microscope (CLSM) and light sheet microscopy are often used. Recent advances in sample clarification methods have enabled us to image the 3D distribution of molecules in deep regions of various specimens^13,14^. However, as the clarifying reagent becomes more effective, the specimen becomes more transparent, making it difficult to obtain anatomical data of the specimen.

We previously developed a 3D imaging method based on the block-face imaging technique and named it “correlative microscopy and block-face imaging” (CoMBI)^15^. Our CoMBI method differs from previously reported block-face imaging methods in its ability to obtain not only serial block-face images but also sections as required. The CoMBI system, which we refer to as CoMBI-C (CoMBI system using a cryostat) in this report, utilizes a standard cryostat for slicing a frozen block, and acquires serial block-face images as 3D anatomical data. The cryostat used for CoMBI can also function as usual to collect sections as required, while pausing block-face imaging. Then, the sections can be stained with various stains as is usually done, and used for microscopic analyses. Thus, our CoMBI method can yield both 3D anatomical data and 2D microscopic data from a single specimen. The correlation between the section and corresponding block-face tells us the location where the section originated in the specimen, which facilitates the interpretation of 2D microscopic images. This correlation can add molecular data to the 3D morphological data, although the molecular data are limited to 2D distribution. To date, the CoMBI-C system has been used in various research fields, such as 3D analysis of primordia of beetle horn^16^, 3D distribution analysis of myelinated fibers of human facial nerves^17^, 3D morphological analysis of polycystic kidney of knockout mice^18^, and 3D visualization of regenerating bones and cartilage of the axolotl jaw^19^. Through these collaborative studies and ongoing collaborations, we found that users of the conventional CoMBI-C system desired compatibility with not only frozen specimens but also paraffin-embedded specimens. In addition, users requested a higher magnification than the 1 × magnification of the conventional CoMBI-C system. To meet these requests, we have developed a new CoMBI system using a sliding microtome (CoMBI-S). The CoMBI-S system can be used for both paraffin-embedded and frozen specimens. The magnification ability of the CoMBI-S system has improved to the point where it yields block-face images with a pixel size of less than 1 µm, which is about five times higher than the conventional CoMBI-C system. In order to be compatible with paraffin-embedded specimens, the transparency of the paraffin needed to be reduced. With the original paraffin transparency, there was an issue in which we could see not only the specimen exposed on the cutting surface but also the deep region of the specimen, resulting in an unclear 3D image. To solve this problem, we have also developed methods to make the paraffin block opaque, which allows us to capture only the specimen exposed on the cutting surface, and improves the visualization of surface structures of the specimen in 3D. Furthermore, we have introduced tannic acid as a contrast enhancer for block-face imaging to improve the visibility of the internal structures of specimens, especially frozen specimens. Here, we describe our new CoMBI-S system and sample preparation methods, and present correlative 2D microscopic images and 3D datasets of various biological specimens.

## Results

### CoMBI system using a sliding microtome; CoMBI-S

**Figure 1:**
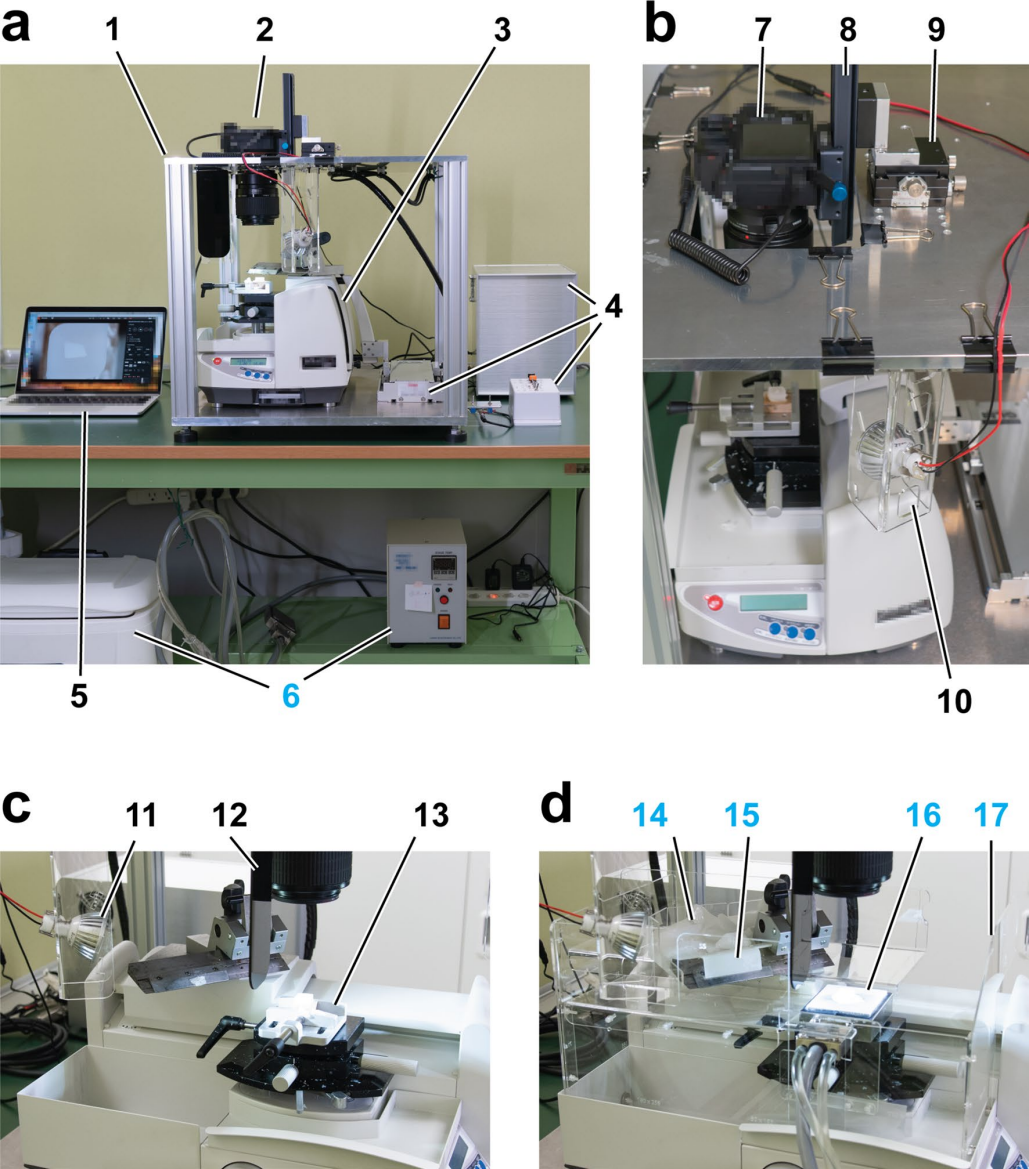
CoMBI system using a sliding microtome. CoMBI-S. (**a**) The front view of the whole system. The aluminum frame (1) consists of top and bottom plates and four pillars. Camera (2) is attached to the top plate. The sliding type microtome (3) is placed on the bottom plate and its handle is attached to a linear motor (4, left). A microcontroller in the box and switches regulate the linear motor and camera shutter release (4). A laptop computer is used for monitoring the block face and storing images (5). In case of frozen specimen, thermoelectric controller and ice water container are used for cooling a specimen holder (6). (**b**) The top view of the system. Camera (7) is attached to the top plate via focusing rail for Z positioning (8) and XY positioning stage (9). Two LED lamps at the front and back of specimen illuminate the block face diagonally (10, 11 in **c**). (**c**) The side view of the setup for paraffin-embedded specimens. The shading plate (12) and specimen holder (13) are shown. (**d**) The side view of the setup for frozen specimens. Dry ice for cooling knife (14), frost distributor (15), specimen holder cooling by Peltier element (16), and plastic walls for keeping the cutting environment cool (17) are shown.

The CoMBI method was designed to obtain both serial block-face images as a 3D dataset and sections as required from a single specimen. We have developed a CoMBI system that is compatible with both paraffin blocks and frozen blocks, and is capable of imaging at a higher magnification than the previous model. Since the new system consists of a sliding microtome, we named it the CoMBI-S system. The main components are a sliding-type microtome with or without a freezing unit, a digital camera with a macro lens, a custom-made metal frame, a liner motor, and LED lights (Fig. 1a,b, Suppl. Fig. S1, Table S1).

At the beginning of development, we selected a sliding-type microtome among various types of microtomes. The sliding microtome achieves slicing by sliding the knife horizontally and by elevating the specimen table toward the knife. Thus, the XY position of the specimen is unchanged, and the height of the block face is always in the same plane. Such behavior is ideal for imaging block faces at higher magnification without blur.

The camera was attached to the top of a metal frame to capture the block face from right above the specimen block. The microtome handle was motorized using a linear motor. The camera shutter release and the motorized handle were regulated by a microcontroller for capturing block-face images automatically every time the microtome slices the block (Suppl. Movie S1). Use of the robust metal frame increases the stability of the system, and automation of slicing and shutter release reduces human error. These components contribute to completion of serial imaging without shift or blur even at the highest magnification (5×). Even if the frame and motor drive are replaced with a consumer camera tripod and manual control, respectively, the system can be constructed at a low cost (Suppl. Fig. S2). In fact, this low-cost system was a prototype of our system and can achieve 3D imaging at lower magnifications (up to 3×).

The distance between the block and the lens must be large enough to make the system capable of slicing the block and collecting the sections. A minimum distance of 40 mm is required for the knife to run between the lens tip and the block. There are many macro lenses having a magnification of 1×, and some macro lenses having higher magnifications of up to 5×. Among them, we chose some lenses for CoMBI-S and listed them in Suppl. Fig. S1 and Suppl. Table S1. These lenses have a working distance (the distance between the lens tip and the object in focus) of more than 40 mm at any magnification, and they provide enough space for collecting sections. A scene showing the collection of a section is shown in a movie (Suppl. Movie S1). For later correlation of sections and block faces, the sections should be stored with the filename of the corresponding block-face image.

The block face is diagonally illuminated by two LED lights, which are located 18 cm away from the block with an adjustable angle between 10° and 40° (Fig. 1b,c). Such positioning of lights affords sufficient space for slicing and collecting sections. The lower the angle of the LED, the more shadows tend to be created between the structures within the specimen. We usually set the angle at 20° to capture images with sufficient brightness and better contrast, though the optimal angle should be determined depending on each experiment.

The sliding microtome that we used here is compatible with paraffin blocks with the default setup, and also with frozen blocks by equipping the optional freezing unit (Fig. 1c,d). The dual compatibility is the advantage of the new system as compared to the previous system, which utilizes a cryostat and is compatible only with frozen blocks^15^. When converting from a setup for paraffin blocks to a setup for frozen blocks, the stage is replaced with a freezing unit, and additional components are attached to keep the slicing environment at a low temperature and to keep the block face clean (Suppl. Movie S2). Among the additional components, the frost distributor is essential and is responsible for keeping the block face clean. It creates a flow of frost from dry ice to both sides of the knife and prevents frost from falling on the block surface (Suppl. Fig. S3a–d). Without the distributor, frost will appear as many white spots on the block face, which is seen as image noise (Suppl. Fig. S3e–g).

**Figure 2:**
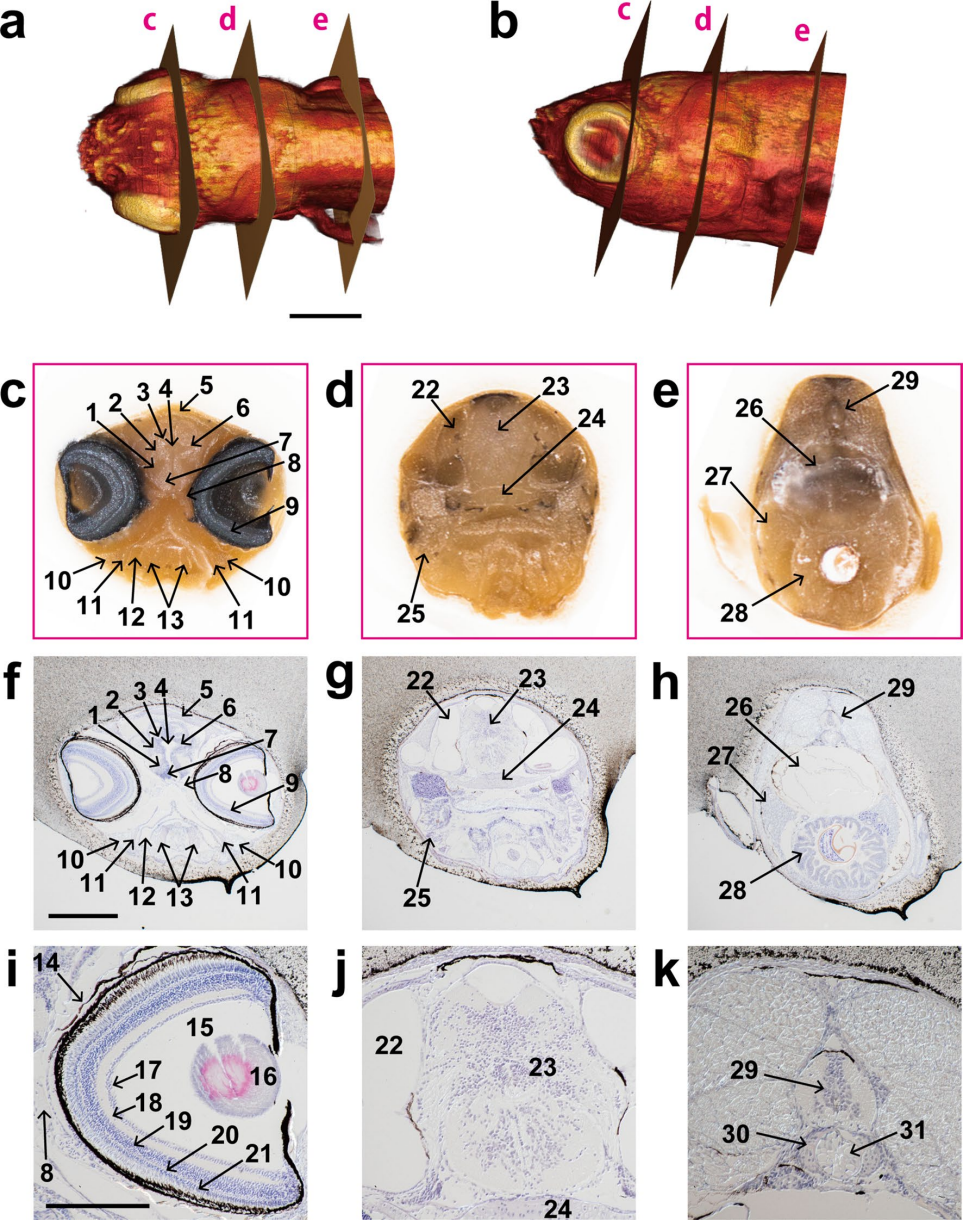
Correlation between 3D image and sections of juvenile zebrafish. A juvenile zebrafish (30 dpf) is sliced at a thickness of 5 µm using CoMBI-S. (**a**, **b**) 3D volume-rendered image using 568 block-face images. (**c**–**e**) Examples of block-face images. (**f**–**h**) Sections were collected at the positions corresponding to (**c**–**e**), and stained with hematoxylin and eosin. (**i**–**k**) Higher magnification of (**f**–**h**). 1: lateral forebrain bundle, 2: ventral thalamus, 3: dorsal thalamus, 4: telencephalic ventricle, 5: habenula, 6: zona limitans intrathalamica, 7: preoptic region, 8: optic nerve, 9: retina, 10: mandibular cartilage, 11: palatoquadrate ceratohyal, 12: pharynx, 13: quadrate, 14: cornea, 15: vitreous body, 16: lens, 17: retinal ganglion cell layer, 18: inner plexiform layer, 19: inner nuclear layer, 20: outer plexiform layer, 21: outer nuclear layer, 22: inner ear, 23: brainstem, 24: cranial base, 25: gill, 26: gas bladder, 27: liver, 28: intestine, 29: spinal cord, 30: vertebral body, 31: notochord remnant. Bars in (**a**), (**f**) 500 µm and (**i**) 250 µm.

**Figure 3:**
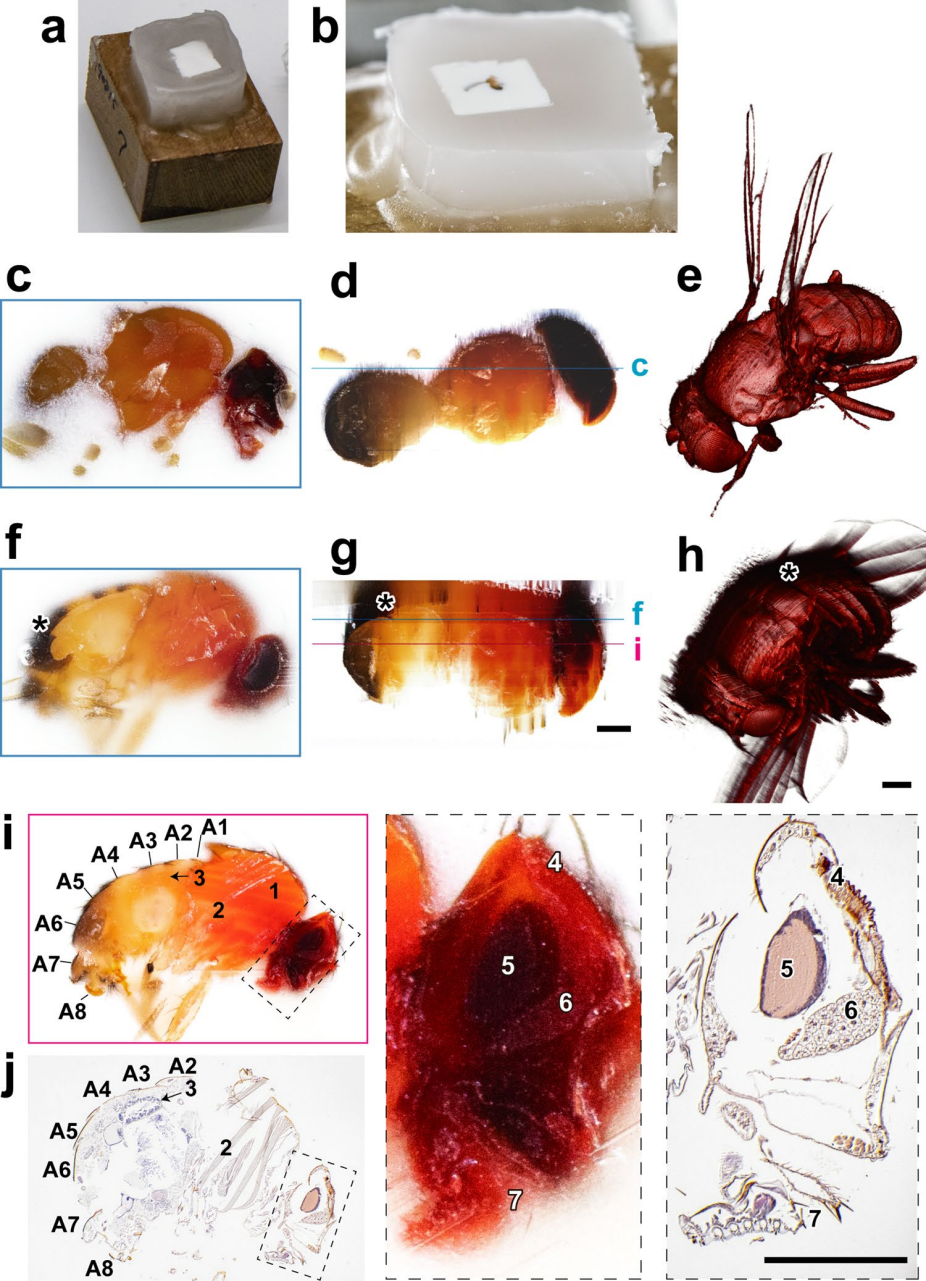
Opacification of paraffin blocks using white agarose gel. Specimens are pre-embedded in agarose gel containing white watercolor, then dehydrated and embedded in paraffin. (**a**) A fly was fixed and pre-embedded in white agarose, then dehydrated and embedded in paraffin. (**b**) In the block face, white agarose gives opaque area around the specimen, while pure paraffin is seen as transparent area around the lump of white agarose. (**c**–**e**) A fly pre-embedded in white agarose generated 276 block-face images with 4 µm-interval. A block-face image (**c**), a reconstructed (**d**), and a volume-rendered image (**e**) are shown. (**f**–**h**) Without white agarose, structures underneath the block face are visible through the paraffin in the block-face image (* in **f**), and a reconstructed plane (* in **g**). These structures are represented in the 3D volume-rendered image as a shadow (* in **h**). Images in (**g**) and (**h**) were reconstructed from 351 block-face images with 4 µm-interval. (**i**, **j**) Correlation between a block-face image (**i**) and microscopic image of a section (**j**) were performed using the specimen shown in (**f**–**h**). Boxed areas in (**i**) and (**j**) are magnified in the right panels. A1–A8: Abdominal segments, 1: Dorso-longitudinal muscles, 2: Dorso-ventral muscles, 3: Midgut, 4: Compound eyes, 5: Brain, 6: Head fat body, 7: Labellum. Scale bars: 200 µm.

**Figure 4:**
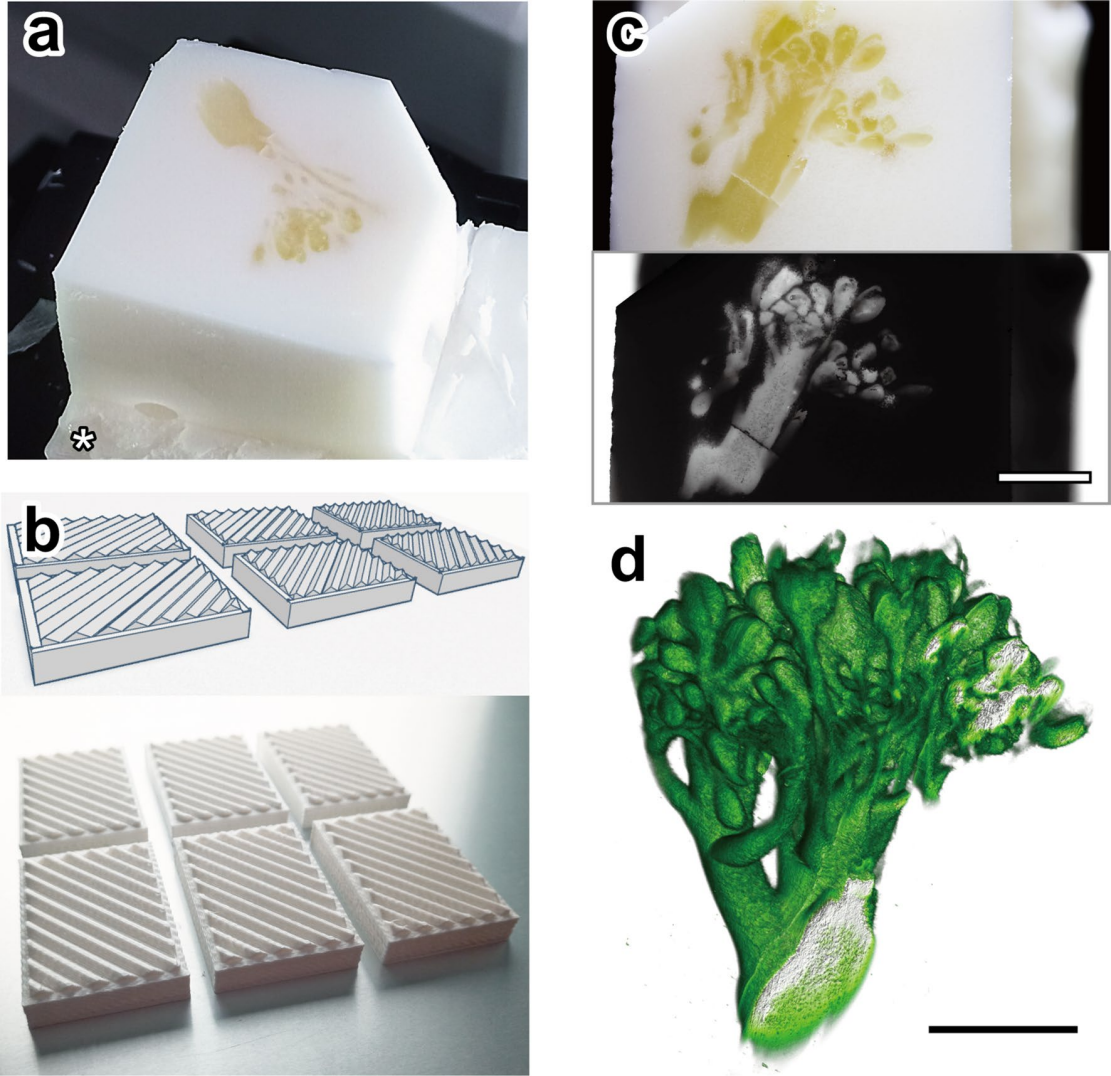
Opacification of paraffin block using white crayon. (**s**) A piece of broccoli was embedded in paraffin containing 6.25% w/w white crayon and placed on the custom-made attachment (asterisk). (**b**) The custom-made white attachment was designed (upper image) and made using 3D printing machine (lower image). Characteristics of the attachment are the white color and the sawtooth pattern on the top, which avoid shadows and increase the area for attaching paraffin block. (**c**) The border between broccoli specimen and white paraffin is apparent on the block face (upper image). When the block-face image was converted to grayscale image for volume rendering, only the cutting face of the broccoli specimen is seen (lower image). (**d**) The serial block-face images (270 images with a voxel size of 20 × 20 × 20 µm) were converted to grayscale images (**c**), then used for volume rendering (**d**). Bars: 5 mm. The STL file of attachments is available at GitHub (https://github.com/combi-3d/CoMBI-Sliding-microtome).

**Figure 5:**
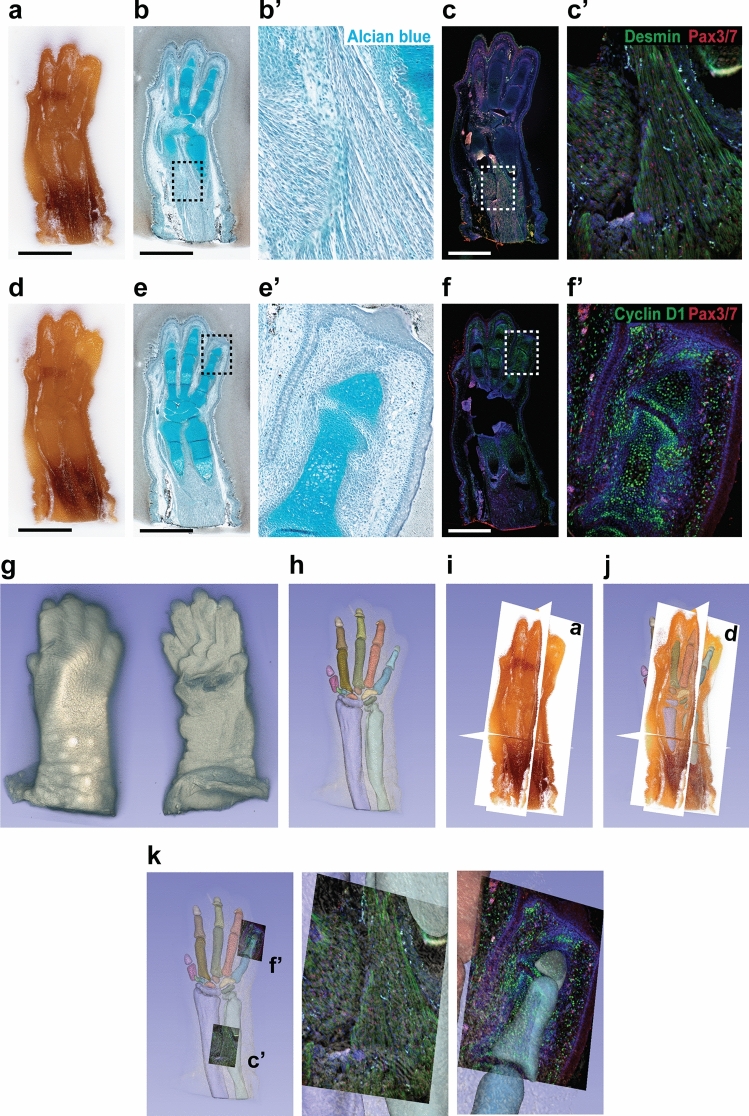
Usability of CoMBI data. Mouse forelimb on E16 were pre-embedded in white-agarose and embedded in paraffin. The 267-serial block-face images with 6-µm interval were obtained. (**a**–**f**) After imaging the 2 block faces shown in (**a**) and (**d**), 2 serial sections were collected and used for Alcian blue staining (**b**, **e**) or immunostaining (**c**, **f**) for desmin (green in **c**), cyclin D1 (green in **f**), Pax3/7 (red in **c**, **f**). Nuclear DNA was labeled with DAPI (blue in **c**, **f**). Boxed areas in (**b**, **c**, **e**, and **f**) are shown at higher magnification (**b′**, **c′**, **e′** and **f′**). (**g**) Volume-rendered image. (**h**) Segmentation of cartilages. (**i**, **j**) Orthoplanes. Planes labeled with (**a**) and (**d**) are identical to block faces shown in (**a**) and (**d**). Segmented cartilage and 3D image by volume rendering are also displayed (**j**). (**k**) Fluorescent micrographs in (**c′**) and (**f′**) are shown in the original location. Bars: 1 mm.

**Figure 6:**
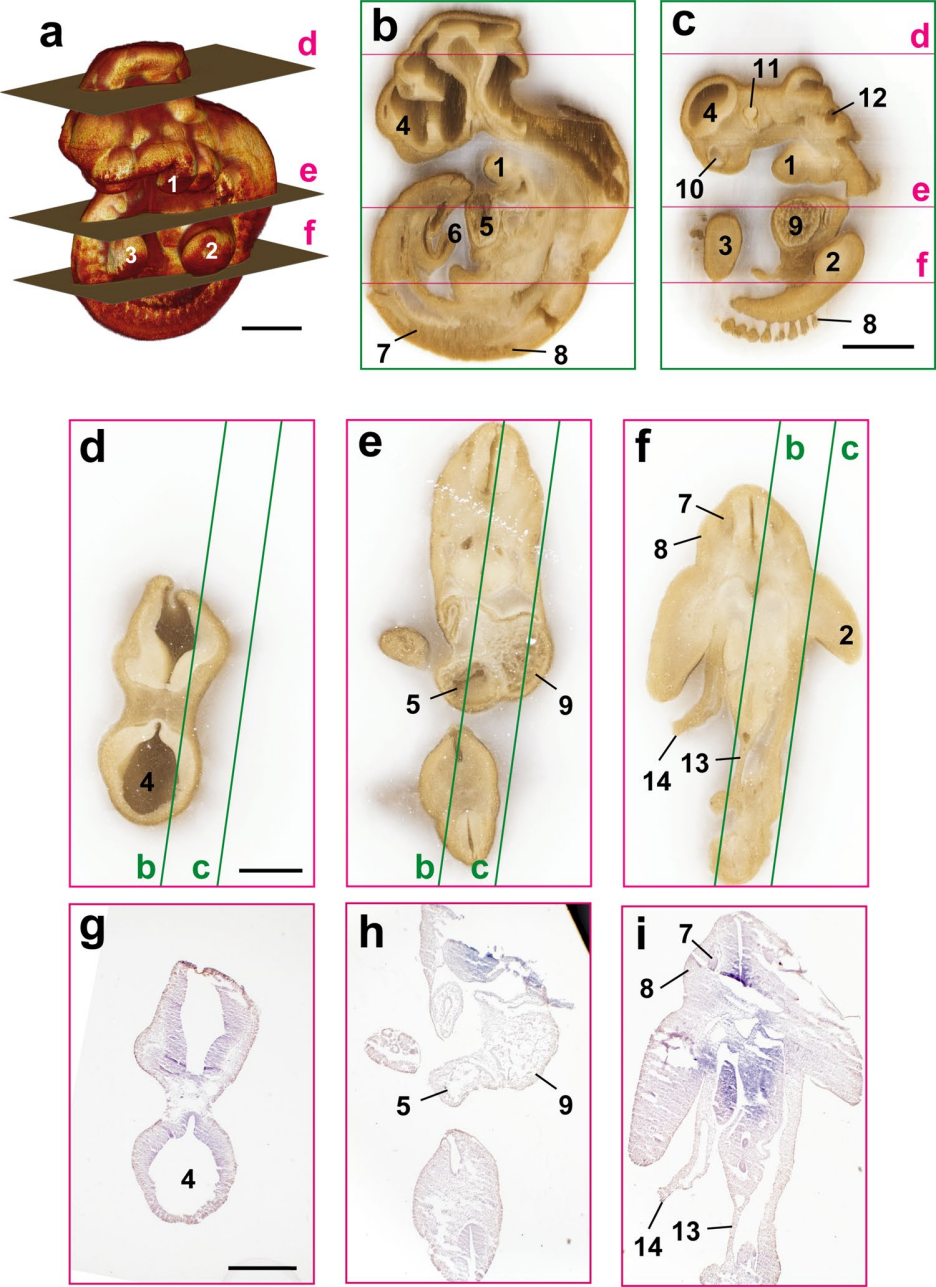
Correlation between 3D image and sections of mouse embryo on E10 in a frozen block. The mouse embryo on E10 was frozen and sliced at a thickness of 10 µm using CoMBI-S system. (**a**) 3D volume-rendered image using 520 block-face images shows the surface structures of the mouse embryo. (**b**, **c**) The reconstructed sagittal planes. (**d**–**f**) Three examples of block-face images are shown. Green lines indicated the positions of (**b**) and (**c**). (**g**–**i**) Sections were collected at the positions of (**d**–**f**). H&E-stained sections can be correlated with block-face images in (**d**–**f**), and the positions where the sections were obtained are indicated as planes in (**a**). 1: Mandibular arch, 2: Forelimb, 3: Hindlimb, 4: Telencephalon, 5: Right ventricle, 6: Umbilical cord, 7: Dorsal root ganglion, 8: Somite, 9: Left ventricle, 10: Nasal pit, 11: Eye, 12: Otic vesicle, 13: Dorsal mesentery of hindgut, 14: Abdominal wall (broken). Bars: 1 mm (**a**, **c**), 500 µm (**d**, **g**).

**Figure 7:**
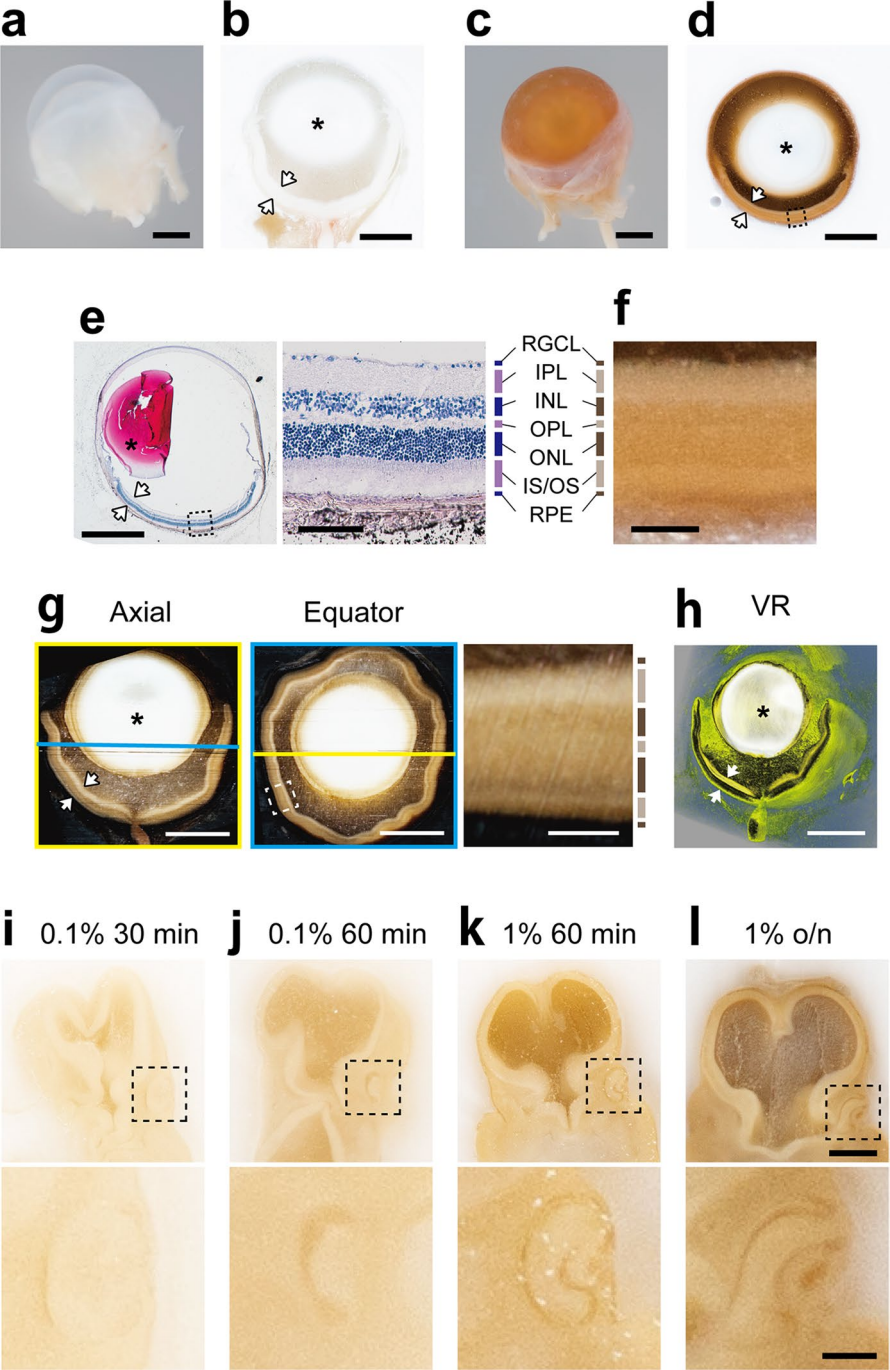
Tannic acid staining improves the visibility of tissues in the frozen blocks. (**a**–**d**) The eyeballs of adult ICR mouse were frozen and sliced for block-face observation. Without tannic acid staining, the retina (arrows) and lens (asterisk) are seen in white (**a**, **b**). By pre-staining with 1% tannic acid for 2 h, the retina is seen in brown with layered pattern, and the lens remains in white (**c**, **d**). The anterior and posterior spaces to the lens (aqueous and vitreous chambers) are seen in darker brown, which may reflect the remaining tannic acid solution or the shadow. (**e**, **f**) A section corresponding to the block face in d was stained with H&E, and used to identify the retinal layers; the retinal ganglion cell layer (RGCL), the inner plexiform layer (IPL), the inner nuclear layer (INL), the outer plexiform layer (OPL), the outer nuclear layer (ONL), inner and outer stripe of the photoreceptor layer (IS/OS), and retinal pigment epithelium (RPE). (**g**, **h**) The eyeball of adult C57BL5 mouse was imaged using CoMBI-S, and generated 919 block-face images with a voxel size of 2 × 2 × 4 µm. The reconstructed axial and equator planes (**g**) show retinal layers. The yellow and cyan lines indicate the positions of reconstructed axial and equator planes, respectively. 3D volume-rendered image (**h**) also shows the retina with some layers (arrows), and the lens (asterisk). (**i**–**l**) Mouse embryos on E10 were stained with tannic acid with indicated concentrations and staining times, then frozen. The block-face images shows that the visibility of tissues in the head region was improving, as increasing the concentration and staining time. The developing eyes in the upper panel (boxed areas) are enlarged in the lower panel. Bars in (**a**–**h**) 1 mm for whole eyeball, and 100 µm for magnified images, (**i**–**l**) 500 µm in the upper panel, 100 µm in the lower panel.

**Figure 8:**
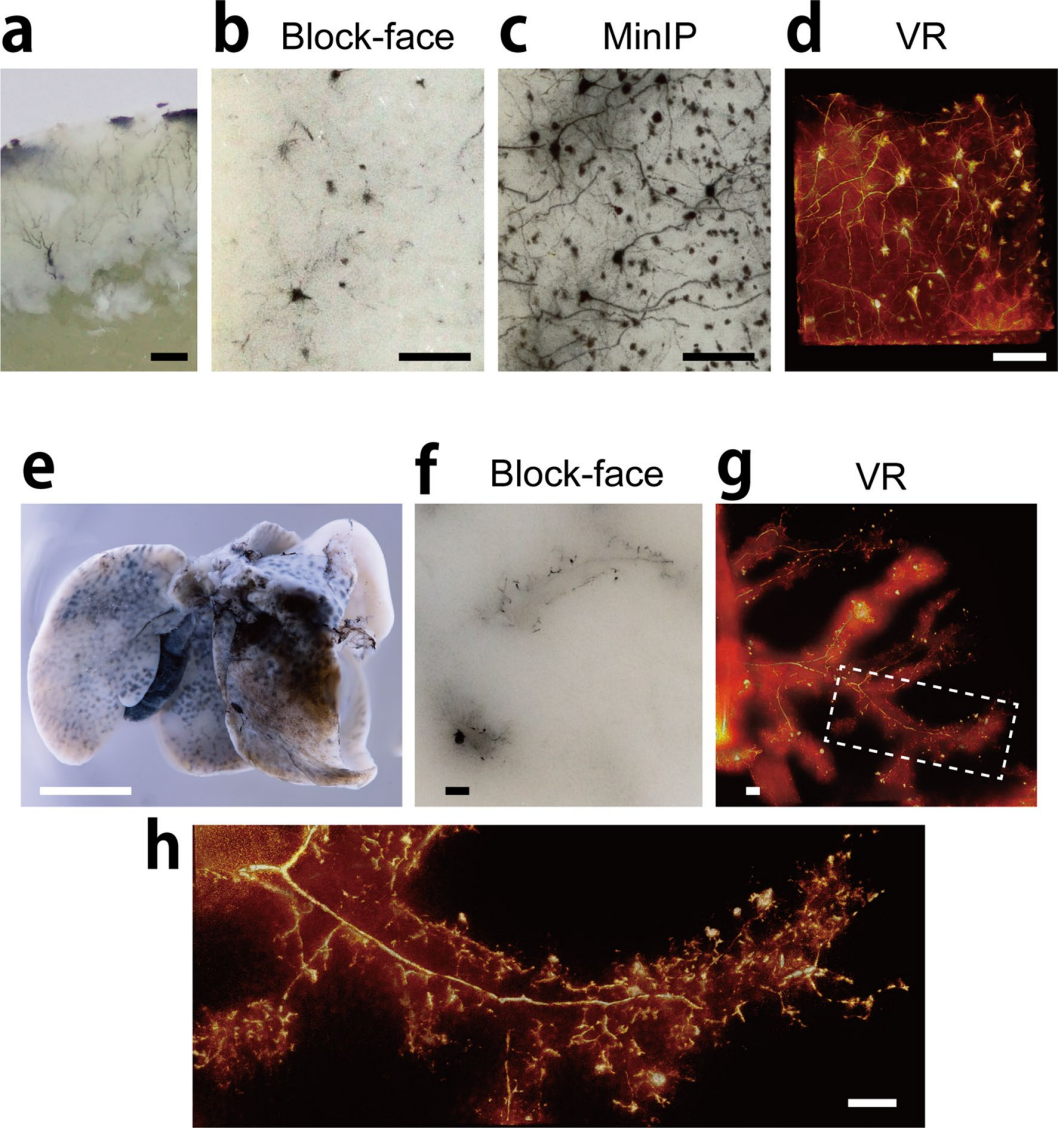
Fine 3D imaging at cellular level. (**a**) Mouse cerebral cortex was stained by Golgi silver impregnation method. (**b**) Block-face image of the stained cortex. (**c**, **d**) The minimum intensity projection (MinIP) image and volume-rendered image were generated from 201 block-face images with voxel size 0.9 × 0.9 × 1 µm. (**e**) Mouse liver were injected with black ink via bile duct. (**f**) A piece of the liver were imaged using CoMBI-S, and a block-face image is shown. (**g**, **h**) 3D volume-rendered images using 401 block-face images with voxel size of 4 × 4 × 1 µm (**g**) or 1 × 1 × 1 µm (**h**) shows the biliary tree. Bars: 100 µm (**a**–**d**, **f**–**h**), 5 mm (**e**).

The following figures indicate the magnification capacity (Figs. 2 and 8) and the compatibility with both paraffin-embedded specimens (Figs. 2, 3, 4, 5) and frozen specimens (Figs. 6, 7, 8), using various specimens: zebrafish (Fig. 2), fruit fly (Fig. 3), broccoli (Fig. 4), and mouse (Figs. 5, 6, 7, 8). In addition, we developed sample preparation methods for improving the quality of block-face images and 3D images, and they are described in Figs. 3, 4, and 7. The conditions for specimen preparation, imaging, image processing, and 3D reconstruction are summarized in Suppl. Table S2.

### Magnification capacity of CoMBI-S

We applied CoMBI-S to juvenile zebrafish embedded in a paraffin block, and imaged the block faces at 5×, the system’s highest magnification (Fig. 2). The zebrafish were 1.5 mm in width, which is about the smallest specimen we observed in this report. The head of the zebrafish was visualized by volume rendering using serial block-face images (Fig. 2a,b). At each of the three locations indicated as c–e in Fig. 2a, we conducted both imaging a block face and collecting a section, and recorded the correspondence between the sections and the block-face images. The sections were stored with the file name of the corresponding block-face image and then stained with H&E for microscopic observation (Fig. 2f–k). Interpretation of structures in a block-face image may be difficult if the only information available is the block-face itself (Fig. 2c–e). When there is a correlation between the block-face image and the microscopic image of the section, the interpretation becomes much easier (Fig. 2c–k). Conversely, when interpreting a section, it is sometimes difficult to interpret the section by itself because of its distortions and the loss of 3D information of the original specimen. Using our CoMBI method, we can determine the degree of distortion of the sections and know where the sections came from in the specimen, making it easier to interpret the sections.

The juvenile zebrafish used here were pre-stained with tannic acid and pre-embedded with white agarose before paraffin embedding to improve the quality of block-face images and 3D images. Details of these sample preparation methods are described in the following figures (Fig. 3, Suppl. Figs. S4,S5).

### Opacification of paraffin blocks using “white agarose pre-embedding method”

Due to the transparency of paraffin, the block face of regular paraffin blocks shows not only the cutting face of the specimen but also the structures in the deep region of the specimen, which is seen through the paraffin. Such structures become undesirable signals in the 3D dataset and interfere with the 3D reconstruction process. Thus, the paraffin needs to be opacified before block-face imaging of paraffin-embedded specimens. We have established two methods for making opaque paraffin blocks: one method is the pre-embedding method using white agarose (Fig. 3), and the other is opacification by adding crayon (Fig. 4). In addition, we designed a custom-made attachment placed between the paraffin block and the microtome stage to ensure the whiteness of the background of the block-face images (Fig. 4).

In the white agarose pre-embedding method, the fixed specimen is first embedded in agarose containing white watercolor paint (Suppl. Fig. S4). Then, the specimen with agarose is fixed again, dehydrated, and embedded in paraffin. We prepared fruit flies in paraffin blocks with or without white agarose pre-embedding, and evaluated the effect on the quality of the 3D images obtained with CoMBI-S (Fig. 3a,b). White agarose is opaque and creates a white area surrounding the specimen and a clear boundary between the specimen and agarose gel on the block-face images (Fig. 3c). Serial block-face images were used for reconstruction of horizontal plane of the fly (Fig. 3d), and volume rendering to show body surface (Fig. 3e). When a fly was imaged without white agarose, the structures in the deep region could be visible through transparent paraffin (Fig. 3f). Undesirable structures like shadows were seen in the reconstructed plane and volume-rendered image (Fig. 3g,h). These data indicate that pre-embedding using white agarose can significantly improve surface observation, but it has limited effect on internal observation. It should be noted that internal observation can be facilitated by correlation with sections (Fig. 3i,j).

There is another advantage of agarose pre-embedding (Suppl. Fig. S4). White agarose embedding and later fixation make any specimen into a rectangular agarose block with a certain degree of hardness. Such agarose blocks can be handled more easily, and it allows us to embed the specimen in paraffin with the desired orientation and determine the slicing direction. We prefer to use white agarose for small or irregular-shaped specimens like zebrafish (Fig. 2) and fruit flies (Fig. 3).

### Opacification of paraffin block using “crayon method” and custom white attachment

Another opacification method is adding crayon directly to paraffin. Broccoli was embedded in paraffin containing 6.25% w/w white crayon and imaged using CoMBI-S (Fig. 4a). As the slicing and imaging progressed, the block face got closer to the plastic attachment at the bottom, and there was a concern that the color and pattern of the attachment would be seen through. To eliminate such concern, a custom-made attachment was made with a 3D printing machine (Fig. 4b). The characteristics of the attachment are the white color and a sawtooth pattern on the top surface. Such pattern increases the surface area for better attachment to the paraffin block and does not create shadows during imaging since it is aligned parallel with the direction of illumination of the CoMBI-S system. Using a combination of white crayons and custom-made white attachments, we ensured that the area around the specimen can be imaged in white through the block-face imaging process (Fig. 4c). The block face presented only the cutting face of the broccoli, indicating successful opacification. Serial block-face images were converted to grayscale images and then successfully used for volume rendering (Fig. 4d). We prefer to use the crayon method for relatively larger specimens, as shown in Fig. 4. If a small sample, such as the juvenile zebrafish in Fig. 2, is embedded in white crayon-paraffin, it will be difficult to see and locate.

### Usability of CoMBI data

Morphological information from only block-face images is limited, however, the corresponding sections can provide various information about molecules, cells, and tissues, and support to interpret block-face images. In addition to Figs. 2, 3 and 4, which show simple H&E-stained sections, further usage of sections and 3D data is shown (Fig. 5, Suppl. Movie S3). Mouse forelimb on embryonic day 16 (E16) was embedded in paraffin and imaged using CoMBI-S system. At the 2 planes (Fig. 5a,d), 2 serial sections were obtained, and labeled with Alcian blue (Fig. 5b,e) or antibodies (Fig. 5c,f), respectively. Serial block-face images were converted to grayscale images, and used to reconstruct forelimb in 3D by volume rendering (Fig. 5g). Using serial block-face images and Alcian blue stained sections, the cartilaginous phalanges, carpal bones, radius, and ulna in development were segmented and shown in 3D (Fig. 5h). When we drew regions of cartilage on the block face images manually, the stained sections were very helpful to learn and confirm the appearance of cartilage. Serial block-face images can also be used for the reconstruction of orthoplanes in real color (Fig. 5i,j). Finally, the fluorescent micrographs show that the myotubes in maturation distributes in the forearm (Fig. 5c, desmin+, Pax3/7+), and that the cells in proliferation are abundant in the cartilage and mesenchyme (Fig. 5f, cyclin D1+). The original locations of these images are shown with 3D cartilage (Fig. 5k). The variety of data types above indicates that the CoMBI method can be applied to a variety of experiments.

### Using CoMBI-S for frozen blocks

CoMBI-S can also be used for frozen blocks by installing additional components: electro-freezing unit for freezing the specimen block, dry ice for chilling the knife, and acryl walls for keeping the environment cool (Fig. 1d, (Suppl. Movie S2). Mouse embryo on E10 in a frozen block was imaged and shown as correlative 3D images and 2D section images (Fig. 6). Volume rendering using the serial block-face images showed the fine surface structure of the embryo (Fig. 6a). The block-face images (Fig. 6d–f) and the reconstructed sagittal plane (Fig. 6b,c) allowed observation of various organ systems in the organogenesis period, including the cardiovascular (Fig. 6b,c,e), gastrointestinal (Fig. 6b,f), and nervous systems (Fig. 6b–d). Sections were collected on the cover glasses at the positions indicated as d–f in Fig. 6a, and were helpful in interpreting the block surface.

The frozen sections of the embryo presented here were damaged (Fig. 6g–i). This is due to the fact that our system does not have a function to keep the frozen section flat after slicing. In the case of a cryostat, a frozen section can be kept flat by sliding it into the gap between the anti-roll plate and the knife stage. The stability of collecting frozen sections with our system is lower than that with a cryostat, though it is also affected by other factors, such as the slicing thickness and the integrity of the specimen.

### Contrast enhancement of the block-face image

Cells and tissues are usually colorless and seen as white on the frozen block surface, except for erythrocytes and melanocytes. We looked for reagents for improving the visibility of tissues on the block face among a variety of staining reagents that we usually use in the laboratory for light and electron microscopy. We found that tannic acid, which is used to stain specimens for electron microscopy, penetrated well into the specimen, did not leak out into the embedding media, and colored the specimen with a moderate intensity. Figure 7 shows the validation of tannic acid as a contrast agent for block-face imaging using the retina, one of the most transparent tissues in the adult body. The whole eyeball of the albino mouse was pre-stained with tannic acid and washed well in PBS. The specimen was then frozen and its block faces imaged. Without pre-staining with tannic acid, the eyeball was almost entirely white, and most of the components within the eyeball were seen as white on the block face (Fig. 7a,b). In contrast, the eyeball pre-stained with tannic acid was brown and showed the retina with a layered pattern on the block face (Fig. 7c,d). A section corresponding to the block face in Fig. 7d was stained with H&E and correlated with it for identifying each layer of the retina (Fig. 7e,f). The correlation revealed that the block face showed an inner plexus layer (IPL) and inner and outer segments (IS/OS) in brighter brown, and inner and outer nuclear layers (INL, ONL) in darker brown. Although the thinner layers were not obvious on the block face, these data indicate that the tannic acid staining greatly improves the visibility of the retina and helps to interpret the block-face image. The effect of tannic acid can, of course, be seen in 3D images (Fig. 7g,h). We imaged the eyeball of C57BL6 mouse by CoMBI-S and reconstructed the axial and equator planes, which showed some retinal layers (Fig. 7g), and the 3D image of the retina, which showed retinal layers with pseudo colors; IPL and IS/OS in yellow, and INL to ONL in black (Fig. 7h). These indicated that tannic acid improved the visibility of the retina on the block face.

The intensity of colorization of tannic acid can be adjusted by changing its concentration and staining time. We stained the mouse embryos on E10 at various concentrations and times. The mouse embryos on E10 used here had been stored in methanol, which is generally used as a standard stock solution before whole mount in situ hybridization and whole mount immunostaining, to make them opalescent and almost invisible in the block face. When these embryos were stained with tannic acid solution and observed on the block face, the visibility of organs improved depending on the concentration and the duration of staining (Fig. 7i–l). The adjustability of the staining will help researchers find the best condition for each specimen. The effect of tannic acid as a contrast-enhancing reagent is evident in frozen specimens; however, its effect is limited in paraffin-embedded specimens (Suppl. Fig. S5).

### Fine 3D imaging of specific structures in specimens

We used CoMBI-S as a fine and full-color 3D imager for specimens that were marked only for the structures of interest (Fig. 8). Golgi silver impregnation is a conventional method for marking neurons. It is still difficult to acquire 3D datasets of the silver depositions for quantification, though there have been some attempts^20–22^. Using our CoMBI-S system, we obtained 3D data for neurons in the mouse cerebral cortex treated with Golgi silver impregnation (Fig. 8a). On the block face, the silver depositions were seen as black dots in various sizes (Fig. 8b). On the image reconstructed from serial block-face images using minimum intensity projection (MinIP), the silver depositions were seen as the shape of neurons (Fig. 8c). The 3D volume-rendered image could also show the neurons with the cell bodies and protrusions including axons and dendrites (Fig. 8d).

The liver consists of hepatocytes, biliary trees, and capillaries, and is often analyzed morphologically focusing on its regenerative capacity. Injection of carbon ink via the bile duct is a simple technique for marking the lumen of biliary trees in mouse liver^23^. However, quantification of ink-injected biliary trees has been difficult with conventional microscopy. Using our CoMBI-S system, we could obtain the 3D dataset of the biliary tree (Fig. 8e–h). In a single block-face image, stumps of the bile ducts or canaliculi filled with carbon-ink could be seen (Fig. 8f). The 3D volume-rendered image showed lumens of the biliary tree (Fig. 8g) and bile canaliculi (Fig. 8h). These data indicate that our system has the capacity to magnify the structures of interest at the cellular level.

## Discussion

In this report, we described a new type of CoMBI system we developed using a sliding microtome (CoMBI-S), which allows correlation between 2D microscopic images of sections and 3D images reconstructed from serial block-face images. Compared with our previous system, CoMBI using a cryostat (CoMBI-C)^15^, the new CoMBI-S system has higher magnification (up to 5×) for block-face imaging, and has expanded compatibility with both frozen blocks and paraffin-embedded blocks. In addition, we proposed tannic acid as a contrast enhancing reagent for block-face imaging.

Our system can obtain block face images with a resolution of less than 1 μm/pixel. The slice thickness can be set to 0.1 µm at the thinnest, but is often set to thicker, e.g., 1–20 µm in this report. These slicing thicknesses were chosen in order to collect sections and to save the total amount of data (Suppl. Table S2). Slicing thicker may cause a concern about the quality loss of the reconstructed image, but from our experience, the quality of the image can be well maintained if the ratio of the pixel size of the block-face image to the slice thickness is up to 1:2. The amount of data per sample were 3.1–39.5 GB, which included 201–1522 block-face images, and were processed for reconstruction using 56–2058 mega voxels/sample. The amount of data to be handled depends on the computer environment.

To make the serial block-face imaging system compatible with paraffin-embedded specimens, it was necessary to opacify the paraffin since the transparency of the paraffin causes even deep regions of the sample to be seen on the block face. An existing block-face imaging system, episcopic fluorescence image capturing (EFIC) developed by Weninger and Mohum, is compatible with paraffin blocks^24^. They opacified paraffin by adding wax color, an additive for making colored candles, stained the specimens with fluorochromes, and then used a fluorescent microscope to detect the fluorescence signals from the tissue exposed at the block surface. They also developed another block-face imaging system for resin blocks, i.e., high-resolution episcopic microscopy (HREM)^3,25^, which is now commercially available (Indigo Scientific, UK). In the case of HREM, they added fluorochrome to resin and detected tissues as shadows using fluorescent microscopy. Both EFIC and HREM systems can successfully detect morphology of the specimen exposed on the cutting face, and also detect specific molecules labeled with fluorochrome. Compared with these existing systems, our system is characterized by use of a consumer full-color digital camera and macro lens for imaging, and use of a popular white watercolor or crayons for opacification. Our system generates 3D datasets in apparent color and also has extended applicability in combination with other labeling methods, as shown in Fig. 8. In addition, it has the unique feature that sections can be used for microscopic analysis, and for helping interpretation of block-face images (Fig. 5).

We also proposed here that pre-staining with tannic acid improves the visibility of structures on the block face, effectively in the frozen blocks, and slightly in the paraffin block. We have not found any significant adverse effects of tannic acid so far. The only disadvantage of tannic acid is that it seems to weaken nuclear staining, but the degree of weakening is small, and images of hematoxylin and DAPI staining were taken without any difficulty (Figs. 2, 3, 5, 6, 7). Tannic acid had no adverse effect on the binding of the three antibodies used in this report (Fig. 5). Since tannic acid becomes darker with long-term storage, we usually image the specimens immediately after staining. If larger specimens require a longer duration for tannic acid staining, sucrose can be added to the tannic acid solution to prevent over-darkening of the color. We have not yet determined the size limit of specimens that can be stained with tannic acid, but at least 1 cm of mouse testis was sufficiently penetrated by tannic acid together with sucrose and evenly stained with a light brown color (unpublished data). There may be other limitations besides size, for example, the lens of an adult mouse was hardly stained by tannic acid (Fig. 7d,g). The effect of tannic acid needs to be evaluated for each biological sample.

Even though the resolution of the imaging system has been improved, the observable fineness of the structures will be poor if the transparencies of the specimen and embedding media are high, or the color contrast of the specimen is insufficient. We successfully opacified the embedding medias; paraffin (Figs. 2, 3, 4) and OCT compound (Fig. 7g, Suppl. Fig. S3, and see “Methods”), to observe the surface structures in 3D. We also improved the visibilities of internal structures by tannic acid staining, however, its effect is apparent in frozen specimens but very mild in paraffin-embedded specimens (Suppl. Fig. S5). During the paraffin-embedding process, the effect of tannic acid seems to be invalid because the sample is made strongly transparent by the penetration of xylene and paraffin. Although sections (Figs. 2, 3, 5, 6, 7) and coloring (Fig. 8) can help interpretation of block-face images, future efforts will include developing a more effective way to opacify the internal structure of paraffin-embedded specimens.

There have been various block-face imaging systems developed in addition to the systems described above^3,24,25^. Block-face imaging systems for frozen blocks have been developed and released from several research groups. For frozen specimens, Yokota’s group at RIKEN developed a 3D internal structure microscope for frozen biological specimens^26^, and they improved the instruments to widen the imaging target, such as metal materials^27^. Wilson and Roy developed a cryo-imaging device for imaging frozen biological specimens^4^, and they put it on the market (BioInVision, USA). These systems can generate full-color 3D datasets from frozen specimens, but they are not designed for collection of frozen sections. Our current CoMBI-S system, as well as our previous CoMBI-C system^15^, can be used to obtain both 3D datasets and sections from a frozen block, although collection of sections can be performed more stably with the CoMBI-C system. Recently, there have been a number of reports involving self-made block-face imaging systems^28–30^. This may be due to the fact that high-resolution digital cameras and high-performance personal computers for 3D imaging are becoming more accessible. Software for 3D imaging is also becoming more accessible and easier to use^31^. In addition, block-face imaging systems including our CoMBI-S system can be assembled at a low cost compared to other current 3D imaging systems, such as CT and MRI for small animals, and fluorescence microscopy for clarified specimens. The total cost of our system was 2,930,000 yen (US $26,742 in January 2021), including the microtome (1500,000 yen = US $14,359), the custom designed frame and motor (900,000 yen = US $8615), and camera/lens (500,000 yen = US $4786). As a possible cost-saving measure, the custom-designed frame and motor can be replaced with a consumer tripod and manual control, as shown in Suppl. Fig. S2. Therefore, the block-face imaging method, including our CoMBI, can be performed at low-cost and is expected to become more popular along with other 3D imaging techniques. Our CoMBI-S and previous CoMBI-C systems will contribute to expanding the use of block-face imaging, and the correlation between the 3D dataset generated by block-face imaging and the 2D microscopic images of the sections improves the reliability of the morphological data.

## Methods

### Animals and plants

Animal experiments were approved by the Animal Care and Experimentation Committee, Gunma University (#18-001), and performed in accordance with the relevant guidelines and regulations, including AVMA Guidelines for the Euthanasia of Animals 2013 and 2020 and ARRIVE guidelines. Mouse embryos were obtained from pregnant Slc:ICR mice (SLC, Hamamatsu, Shizuoka, Japan). Mice were sacrificed by cervical dislocation under isoflurane anesthesia (Wako Pure Chemical Industries, Osaka, Japan). Embryos were fixed with 3% paraformaldehyde in 0.1 M phosphate buffer (PFA) and stored in methanol at − 30 °C. Dead wild fruit flies were obtained by extermination for public health, using a flytrap (Earth Corporation, Tokyo, Japan). The flies were identified as *Drosophila lutescens*, using DorsoWing software^32^. Zebrafish (*Danio rerio*, AB line) were bred in our laboratory as described previously^33^. Zebrafish on 30 days post fertilization (dpf) were anesthetized with MS-222 (Merck, Dermstadt, Germany) and then sacrificed by rapid chilling on ice. Insects and fish were fixed with Davidson’s fixative containing 4% paraformaldehyde (PFA), 10% acetic acid, and 30% ethanol, and mouse tissues were fixed with 4% PFA in 0.1 M phosphate buffer (PB). Broccoli (*Brassica oleracea var. italica*) was purchased from a grocery store in Tsuruoka-city (Yamagata, Japan). A piece of broccoli was fixed with 3% PFA, 0.2% glutaraldehyde, 0.05 M PB pH6.8, vacuum-treated at 0.01 MPa for 2 h, and dehydrated using a graded series of ethanol and tert-butanol. Some animal specimens were stained with 1% tannic acid (Nacalai Tesque, Kyoto, Japan) in PBS for 1 h–1 day, and washed in PBS for 1 h. The stained specimens were immediately subjected to the embedding process since long-term storage of the specimens in PBS would result in a darkening of the color. When the pre-stained specimens had to be stored for a long time, 10% or less sucrose was added to delay the change in color.

### Microtome

For sectioning paraffin blocks, a microtome (Retratome REM-710; Yamato Kohki Industrial, Saitama, Japan) and disposable knifes (A35; Feather, Osaka, Japan) were used. For sectioning frozen blocks, the microtome was equipped with an electro freezing unit (MC-802A; Yamato Kohki) and a cooling water tank (Marine Ultra Breeze 28 Roller; Igloo Products, TX, USA). The applicable block size varies depending on the space between stage top and knife, the use of accessory spacers for adjusting knife holder height, and the working distance of the lens in use (See Suppl. Fig. S1 and Suppl. Table S1 for details). The maximum height of the paraffin block is 36 mm, while the maximum height of the frozen block is lower than that of paraffin block (about 10–20 mm), because the frozen state needs to be kept stable. Custom-made acrylic parts (walls, dry-ice holder, frost distributer, and lamp holder) were made from 2 mm-thick acrylic plate using a laser cutter (Smart Laser CO_2_, SmartDIYs, Yamanashi, Japan).

### Camera

The block faces were photographed using a digital camera (Sony a7RIII (Tokyo, Japan) or Canon 5Ds R (Tokyo, Japan)) with macro lenses (Suppl. Fig. S1 and Suppl. Table S1). Mount adaptors were used if needed (Sigma MC-11 (Kanagawa, Japan) or Kenko Sony E body-Nikon F lens adaptor (Tokyo, Japan)). It should be noted that when we used Canon's lens with Sony's camera and Sigma's adaptor, imaging was possible, but there was a delay in recognition of the lens at startup. Thus, use of a combination of lens and camera body from the same manufacture, or use of a manual control lens, is recommended. A remote cable (E-6588 (Etsumi, Tokyo, Japan) or 3-pin 2.5-mm audio cable) was connected to the camera for shutter release control. The camera system captures a 36 × 24-mm area at a magnification of 1x, and a 7.2 × 4.8-mm area at the highest magnification. An image has 7952 × 5304 pixels (Sony camera) or 8688 × 5792 pixels (Canon camera); thus, the pixel size can be calculated as 0.8–4.5 µm/pixel. The camera was attached to an order-made aluminum frame (Kiryu DK, Gunma, Japan) via a focusing rail (Castel L Einstellschiten; Novoflex Präzisionstechnik GmbH, Memmingen, Germany). The camera settings used were as follows: shutter speed 1/30–1/125 s, aperture size f/2.8 to f/4, and sensitivity ISO 200 to 2000 (Suppl. Table S2).

### Motor control of microtome and camera shutter release

The handle of the microtome is motorized with a linear motor (GLM10, THK, Tokyo, Japan). For releasing the shutter, the handle is stopped at the rear end for 3 s until the system become stable, and then the transistor triggers the camera shutter release. The motor and transistor were regulated using a PIC microcontroller (Microchip Technology, Chandler, AZ, USA). These motor and camera control system was custom-made (Kiryu Denshi, Gunma, Japan).

### Illumination

The samples were illuminated with two LED lamps (3.8 W, MR-16; Optosupply, Hong Kong), which illuminated the block face evenly and diagonally. The illumination can be adjusted in two levels by selecting 9 V or 12 V power supply.

### Opaque paraffin blocks

White crayon (Crayola, Easton PA, USA) was added to paraffin at a concentration of 6.25% w/w, and melted at 60 °C to mix. Fixed specimens were dehydrated using graded series of ethanol and xylene as usual and mounted in this white paraffin. Otherwise, fixed specimens were pre-embedded in 1% fish gelatin (Merck, Dermstadt, Germany)/0.4% low-melting-point agarose (Nacalai Tesque, Kyoto, Japan)/0.05–0.1% w/v white watercolor paste (Pentel Watercolors F, Tokyo, Japan). The composition of the embedding solution was based on and modified from the pre-embedding method developed by Ushida and colleagues for preserving specimen orientation within a block^34^. The white agarose block containing the specimen was fixed further with 4% PFA in 0.1 M PB for 1 h/mm, dehydrated, and mounted in regular paraffin. Regarding the white agarose-based opacification method, we illustrated the process in Suppl. Fig. S4.

### Frozen blocks

Specimens were mounted in OCT compound (Sakura Finetek Japan Co., Ltd., Tokyo, Japan) and cooled with liquid nitrogen and coolant (2-methylbutane; Fujifilm-Wako, Osaka, Japan). Watercolor paste (Pentel F, Tokyo, Japan) was added to the OCT compound at 5–10% w/w for opacification. We routinely use either black paste (Fig. 7g, Suppl. Fig. S3), or white paste (not used in this report).

### Acquisition of block-face image series and sections

For paraffin blocks, the slice thickness was set at 2–20 µm. Block-face images were automatically captured every slice. Sections were collected as needed while pausing slicing. Sections were picked up with tweezers and floated on warmed water at 42 °C to be expanded. The sections were scooped out with a glass slide (non-coated glass slide for H&E staining, or poly-l-lysine coated glass slide for immunostaining, Matsunami Glass, Osaka, Japan), and dried to be adhered. For frozen blocks, the slice thickness was set at 1–10 µm, and the temperature of the electro freezing unit was set at − 20 to − 15 °C. Frozen sections were collected on poly-l-lysine-coated cover glass (Matsunami Glass). Custom-made acrylic walls were attached around the microtome to keep the cutting environment cool and to avoid frost. Dry ice was put on the knife using a custom-made acrylic holder. When the sections were collected, the file names of the corresponding block-face images were recorded for subsequent correlation analysis. For collecting frozen sections, slice thickness needs to be thicker, for example, 10 µm (Fig. 6). When only fine 3D imaging is performed using frozen blocks without collecting the sections, the slice thickness can be set thinner, such as 1–4 µm (Figs. 7, 8).

### Histochemical staining for frozen and paraffin-embedded sections

Frozen secions were immersed in PBS, while paraffin sections were dewaxed, rehydrated, then immersed in PBS. Hematoxylin and eosin (H&E) staining was performed using Meyer’s hematoxylin solution (Muto Pure Chemicals, Tokyo, Japan) and 1% eosin in water (Fujifilm-Wako, Osaka, Japan). For Alcian blue staining, sections were rehydrated, stained with 1% Alcian blue 8GX (Sigma-Aldrich-Japan, Tokyo, Japan) in 3% acetic acid for 15 min, and stained with Meyer’s hematoxylin solution for 1 min, and 0.1% eosin in DW for 1 min. Microscopic images were obtained using a Nikon AZ-100 (Tokyo, Japan) and a Sony a7RII (Tokyo, Japan).

### Immunostaining for paraffin-embedded sections

Mouse embryo on E16 was fixed with 4% PFA in phosphate buffer overnight, and dehydrated and stored in methanol on − 30 °C until use. The specimen was rehydrated and stained with 1% tannic acid in PBS overnight, pre-embedded with white-agarose, dehydrated again, and embedded in paraffin. For immunostaining, paraffin sections were dewaxed, rehydrated and autoclaved at 120 °C for 15 min in 10 mM citric acid pH6.0 buffer. The sections were blocked with 5% normal donkey serum, and incubated with rabbit anti-desmin (1:100, Cell Signaling Technology #4024S, Danvers, MA, USA), rabbit anti-cyclin D1 (cl:SP4, 1:100, Thermo Fisher Scientific K.K., #RM-9104-SO, Tokyo, Japan), and goat anti-Pax3/7 (1:200, Santa Cruz Biotechnology #sc-7748, Dallas, TX, USA)^35^ for overnight at 4 °C, and followed by AlexaFluor488-donkey anti-rabbit IgG antibody (1:500, Jackson Immunoresearch, West Grove, PA, USA), Rhodamine-RedX-donkey anti-goat antibody (1:500, Jackson Immunoresearch), and DAPI (1:200 of saturated solution) for 1 h at room temperature. All micrographs were obtained using Nikon AZ-100 fluorescent microscope equipped with a CCD camera (SPOT RT3, Diagnostic Instruments, Sterling Heights, MI, USA) or a digital camera (Sony a7RII).

### Image processing

Image processing was performed as described previously^15^. Briefly, images were saved as RAW format files and converted to JPEG using Adobe Bridge (San Jose, CA, USA). Serial images were trimmed to exclude margins. In most cases, images were resized to reduce data amount and to make the resolution number an integer for convenience. For the resizing process, the scale was photographed before or after serial block-face imaging and used as a reference. The color series was saved and used for reconsruction of orthoplanes. Grayscale series was converted from color series and used for volume rendering. Image processing from trimming to grayscale conversion were automatically executed using Adobe Photoshop (action and batch commands), or ImageJ (macro function). OsiriX MD (v11.0.2; Pximeo SARL, Bernex, Switzerland) or Horos (v3.3.6 64-bit; The Horos Project, Purview, Annapolis, MD, USA) were used to convert JPEG images to DICOM images and to reconstruct planes by “Multiple planar reconstruction (MPR)” and 3D images by “Volume rendering (VR)” (Figs. 2, 3, 4, 6, 7, 8). 3D slicer^36^ was also used for 3D visualization, using modules “Volumes” to define voxel size, “Transform” to registrate fluorescent images, “Segmentation” to mark the region of interest, and “Volume Rendering” (Fig. 5). For overlay multiple data, the tool bar of “Views” and module “Volume Rendering” were used to adjust opacities. Computers used were as follows: Mac Pro Late 2013 model (CPU: 3.7 GHz Quad-Core Intel Xenon E5, DRAM: 32 GB 1866 MHz DDR3, graphics: 2 × AMD FirePro D300 2048 MB, and storage: 500 GB SSD), iMac 27 inch 2017 model (CPU: 4.2 GHz Intel Core i7, DRAM: 64 GB 2400 MHz DDR4, graphics: AMD Radeon Pro 580 8 GB, and storage: 1 TB SSD), MacBook Pro 2018 model (CPU: 2.9 GHz Intel Core i9, DRAM: 32 GB 2400 MHz DDR4, graphics: AMD Radeon Pro Vega 4 GB, and storage: 1 TB SSD)or MacBook Pro 2018 model (CPU: 2.9 GHz Intel Core i9, DRAM: 32 GB 2400 MHz DDR4, graphics: AMD Radeon Pro 560X 4 GB, and storage: 1 TB SSD) (Apple Japan, Tokyo, Japan).

## Supplementary Information

Supplementary Information.

Supplementary Video 1.

Supplementary Video 2.

Supplementary Video 3.

## Data Availability

STL file of custom attachments (Fig. 4), Arduino codes (Suppl. Fig. S2) and SVG files of acrylic parts (Suppl. Figs. S3, S4) are available at GitHub (https://github.com/combi-3d/CoMBI-Sliding-microtome).
