# Supplementary material for: Correlative microscopy and block-face imaging (CoMBI) method for both paraffin-embedded and frozen specimens
Source: Sci Rep. 2021 Jun 23;11:13108. doi: 10.1038/s41598-021-92485-5 (PMC8222340; doi:10.1038/s41598-021-92485-5)
Supplement: Supplementary file 1 — Supplementary Information. [file 41598_2021_92485_MOESM1_ESM.pdf]

Supplementary Information

Supplementary Movie S1
Video of the CoMBI-S in operation shows serial block-face imaging, and also section collection while pausing block-face imaging.

Supplementary Movie S2
Time-lapse video shows conversion of CoMBI-S system from a setup for frozen block to a setup for paraffin-embedded block.

Supplementary Movie S3
Usage of data obtained by CoMBI, including volume rendering, segmentation, orthoplanes, and cor relation between 2D microscopic data and 3D images.

Supplementary Figure S1 Lenses available for CoMBI-S.
Working distances (WD) of Canon 65 mm 1-5 x macro lens (a), Laowa 25 mm 2.5-5 x macro lens (b), Nikon 60 mm macro lens (c), and Nikon 105 mm macro lens (d) are shown. WDs (38 - 148 mm) afford enough work space for sectioning, illuminating block-face, and collecting sections. Individual length values of WD are summarized in Suppl. Table S1. We found that electric recognition of Canon lens by Sony camera via Sigma adaptor is delayed for a few minutes, even though most of data in this paper was obtained with the Canon lens. There is no issue for recognition of the manually controlled lens, such as Laowa and Nikon lens.

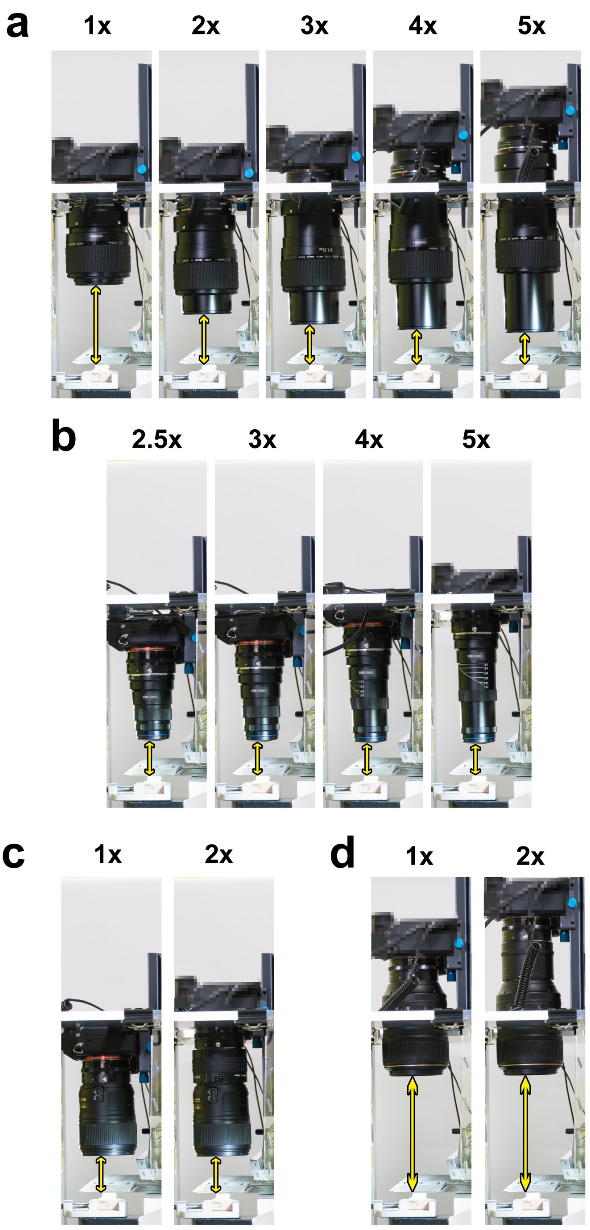

Supplementary Table S1 Working distances of various lens systems (mm)

Lens	Magnifications 1x	2x	2.5x	3x	4x	5x
Canon, MP-E 65mm F2.8 1-5 x Macro Photo	95	60	-	48	42	38
Laowa, 25mm F2.8 2.5-5 x Ultra Macro	-	-	44	43	41	40
Nikon, AF-S MicorNikkor 60mm F2.8G ED	47	47 *				
Nikon, AF-S VR MicorNikkor 105 mm F2.8G IF-ED	146	148 **				

* Kenko 2x teleconverter, ** Nikon 2x teleconverter

Supplementary Table S2 Summary of specimen preparation, imaging, image processing, and 3D reconstruction

Figures	Specimens			Imaging										Image processing						3D Reconstruction				Note
	Source	Block type	Treatment	No. of slicing	Thick-ness (µm)	Camera	Lens	Exp. (sec)	ISO	Aper-ture	Magni-fication	Handle drive	Imaging time *	Format of original blockface image	Amount of RAW or JPEG (GB)	RAW-JPEG conversion time **	Pixel size of original images (µm/pixel)	Pixel size of resized images (X, Y, µm)	Interval for reconstruc-tion (Z, µm)	Applica-tion	No. of images	Image size (pixels)	No. of voxels (mega)	
Fig. 2	Zebrafish	Paraffin	tannic acid	545	5	Sony a7RIII	Canon MP-E 65 mm	1/40	400	F3.2	4.6	motor	1h 30m	RAW, full-size	23.4	09m	0.97	2.5	5	Horos	545	672 × 604	221	
Fig. 3a-e	Drosophila	Paraffin	none	702	2	Sony a7RIII	Canon MP-E 65 mm	1/45	800	F3.5	3.6	motor	1h 01m	RAW, APS-C	12.9	08m	1.25	3	4	Horos	351	719 × 1081	272	Half number of images were used by skipping one image.
Fig. 3f-j	Drosophila	Paraffin	none	276	4	Sony a7RIII	Canon MP-E 65 mm	1/30	1000	F3.5	5.0	motor	43m	RAW, APS-C	5.1	06m	0.9	3	4	Horos	276	924 × 765	195	
Fig. 4	Broccoli	Paraffin	none	370	20	Canon EOS 5Ds	Canon MP-E 65 mm	1/60	400	F3	1.0	motor	32m	JPEG, full-size	3.1	---	4.08	20	20	Horos	370	1100 x 1100	447	
Fig. 5	Mouse E16 limb	Paraffin	tannic acid	267	6	Sony a7RIII	Laowa 25 mm 2.5-5x	1/60	200	F4	2.5	motor	57m	RAW, APS-C	4.9	05m	1.48	3	6	3D slicer	267	1411x810	305	
Fig. 6	Mouse E10	Frozen	tannic acid	523	10	Sony a7RIII	Canon MP-E 65 mm	1/30	1600	F3.5	2.9	motor	1h 17m	RAW, APS-C	9.6	06m	1.54	3	10	Horos	523	1483 × 959	743	
Fig. 7g, h	Mouse eye ball	Frozen	tannic acid	919	4	Sony a7RIII	Canon MP-E 65 mm	1/80	400	F3.2	4.0	manual	43m	RAW, full-size	39.5	3h 23m	1.12	2	4	Horos	919	1600 x 1400	2058	Other data in Fig. 7 are not serial imaging.
Fig. 8d	Mouse brain	Frosen	Golgi's staining	201	1	Sony a7RIII	Canon MP-E 65 mm	1/125	400	F2.8	5.0	motor	11m	RAW, full-size	8.6	1h 26m	0.9	0.9	1	Horos	201	554 x 506	56	
Fig. 8g	Mouse liver	Frozen	Ink injection	1522	2	Sony a7RIII	Canon MP-E 65 mm	1/60	2000	F3.5	5.0	manual	2h 12m	RAW, APS-C	28.1	52m	0.9	4	2	Horos	1522	1162 x 775	1370	
(Fig. 8h)	Mouse liver	Frozen	Ink injection	1522	2	Sony a7RIII	Canon MP-E 65 mm	1/60	2000	F3.5	5.0	manual	2h 12m	RAW, APS-C	28.1	52m	0.9	1	2	Horos	384	1900 x 924	674	A part of the image series in Fig. 8g were used for reconstruction.

* Imaging time includes time for collecting sections.
** RAW-JPEG conversion time basically depends on the CPU's capability. However, memory occupancy also affects the conversion time, which varies depending on the situation.

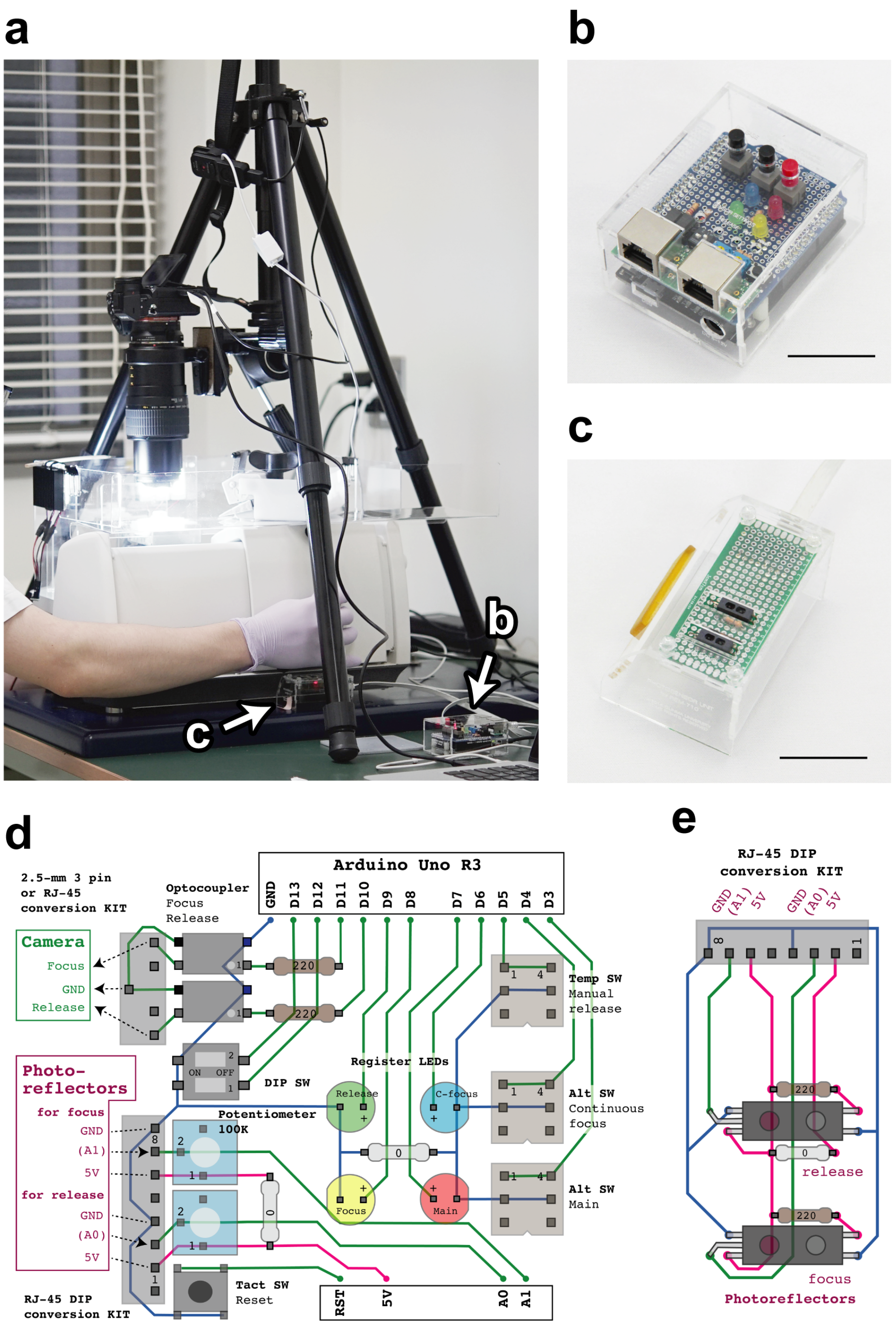

Supplementary Figure S2 Low-cost type of CoMBI-S.

a: The handle is controlled manually, and camera is set on the commercially available tripod (Husky #1003; Toyo Trading, Kyoto, Japan) instead of the handle motor and aluminum camera frame which is shown in Fig. 1. Camera shutter is regulated by a microcontroller (Arduino) which sense the handle position by an infrared-reflective sensor, and trigger shutter release by an optocoupler. Both block-face imaging and section collection can be performed using this system, however, the suitable magnification is up to 3x. At a higher magnification such as 3-5x, block-face images could be blurred due to the instability of the system. The instability of this low-cost system is due the own weight of moving handle, operator’s movement, and instability of tripod. b: Controller unit including Arduino. c: Sensor unit. d, e: Circuits of controller and sensor units. Bars in b, c: 3 cm. Arduino codes are available at GitHub (https://github.com/combi-3d/CoMBI-Sliding-microtome).

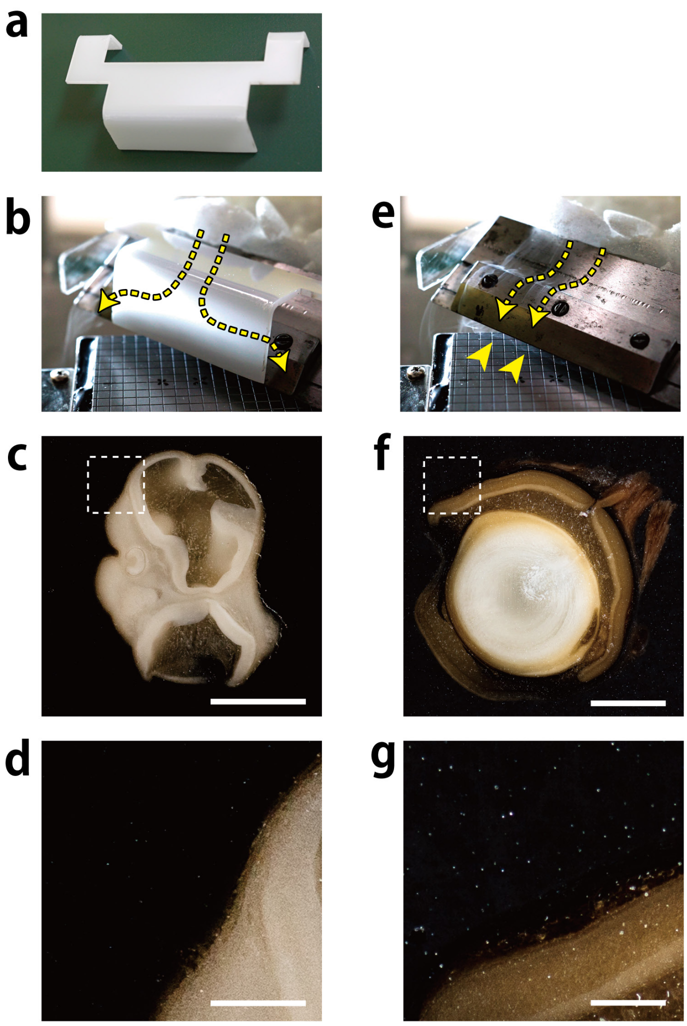

Supplementary Figure S3 Frost distributor for keeping the block-face clean. a - d: The frost distributor was made of an acrylic plate (a), and set on the knife holder (b). Frost derived from dry ice flows to both sides (arrows), and does not fall on the block-face. Using the frost distributor, less frost is found on the block face. e - g: Without the frost distributor, frost flows on the slope of knife (arrows in e) toward the specimen stage (arrowheads in e). Note that the block-face image shows many white dots of frosts (f, g). Bars: 1 mm (c, f), 200 µm (d, g). The SVG file of frost distributor is available at GitHub (https://github.com/combi-3d/CoMBI-Sliding-microtome).

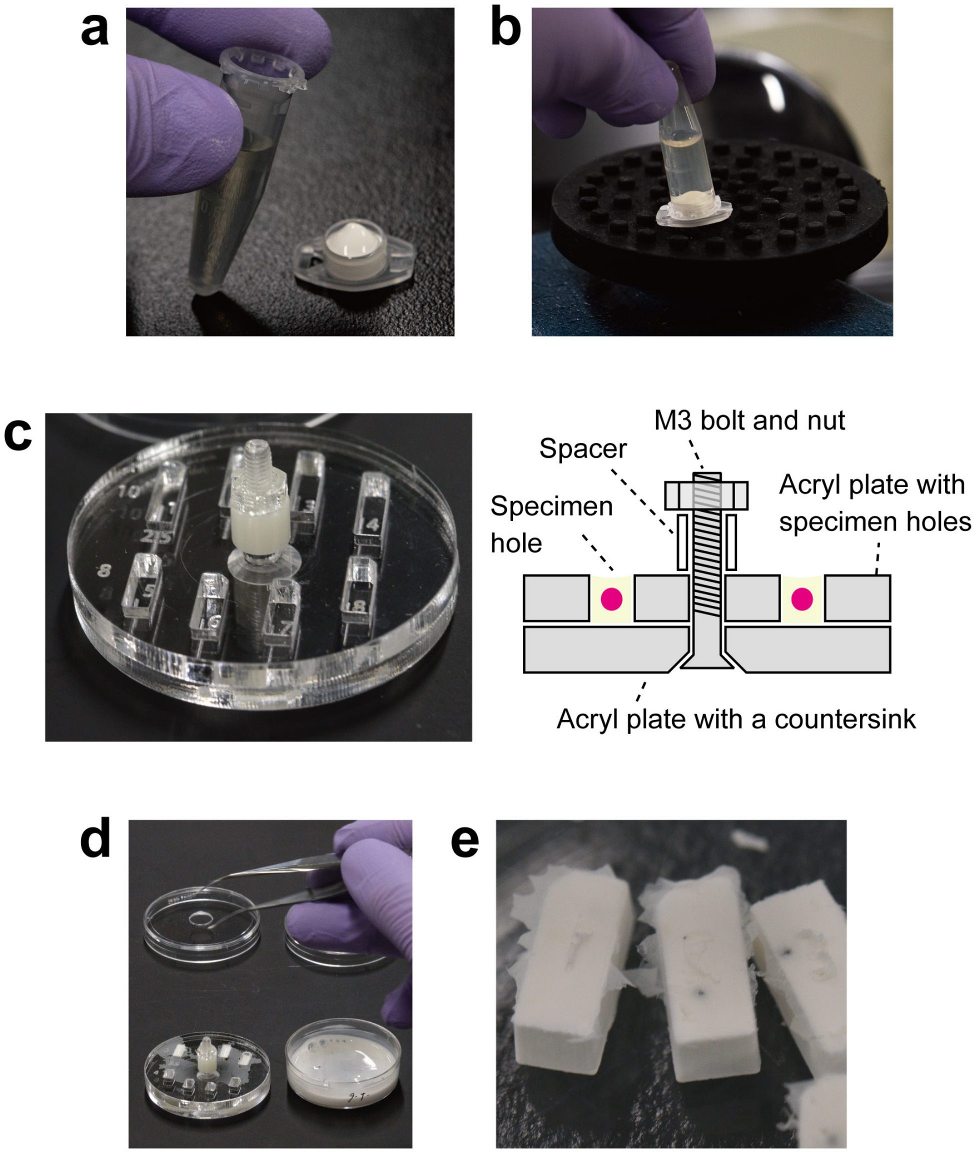

Supplementary Figure S4 Procedure for pre-embedding with white agarose.
a, b: Prepare two solutions; 1 ml of 5% fish gelatin/DW containing 0.3 – 0.5 g of white watercolor paste, and 0.5% low-melting point agarose in DW. Gelatin solution and watercolor paste are taken in 1.5-ml tube and its cap, respectively, and mixed well by a vortex mixer. c: Prewarm the mold for agarose embedding at 42 ºC. The mold was made of acryl plates, and the size and shape of the specimen holes can be customized to suit for each specimen. d: Prepare mounting media by mixing 5% fish gelatin with white watercolor and 0.5% agarose in the ratio 1:4 at 42 ºC. Pour mounting media into the hole. When placing the sample, the orientation of the sample should be noted. Cool the mold to 4 ºC for more than 30 min. e: Fix the agarose block with 4% paraformaldehyde for 1 hour/mm. After the fixation, the agarose block becomes relatively hard and easy to handle. Regardless of the shape of the specimen, the agarose block will always become rectangular, which allow us to determine the orientation when embedding in paraffin. The SVG file of molds is available at GitHub (https://github.com/combi-3d/CoMBI-Sliding-microtome).

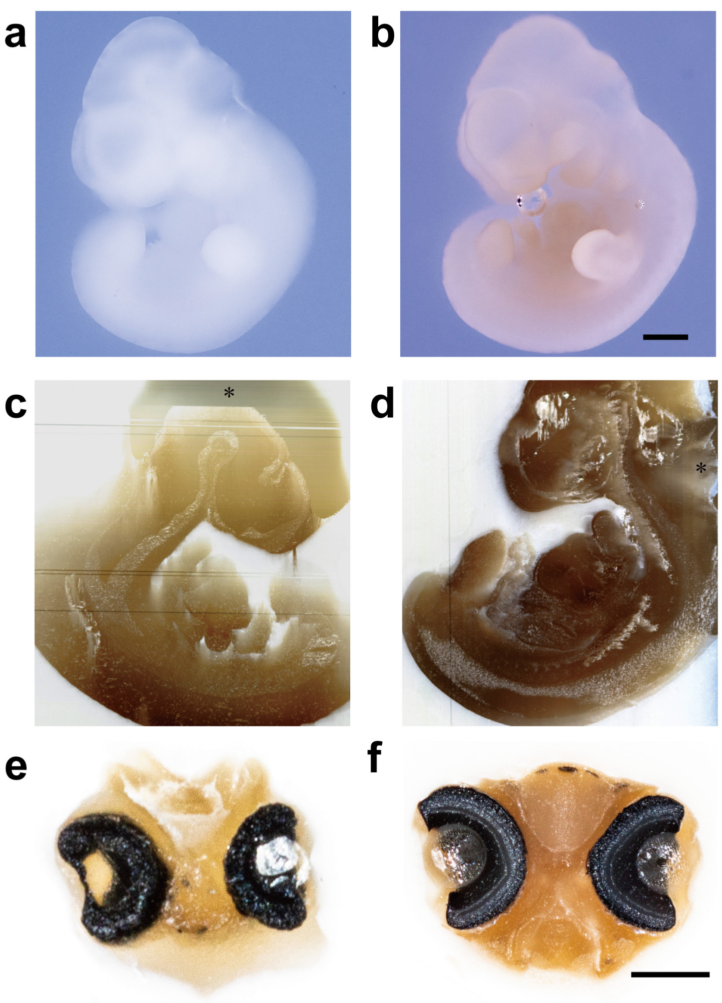

Supplementary Figure S5 Effect of tannic acid staining is limited for the paraffin-embedded specimens.
Mouse embryos on E10 and zebrafish on 30 dpf were stained with 1 % tannic acid for overnight. a, b: Macroscopic images of unstained (a) and tannic acid-stained (b) mouse embryos in PBS. c, d: Mouse embryos embedded in paraffin were imaged by CoMBI-S system. Sagittal planes ware reconstructed from 386 (a) or 314 (b) block-face images. e, f: Block-face images of unstained (e) and tannic acid-stained (f) zebrafishes. By tannic acid staining, specimens in the block-face image and reconstructed image are seen in darker brown, however, the visibility of internal structures was almost unchanged. The zebrafish specimen in f is also presented in Fig. 2. Bars: 500 µm.
